# Bilateral Multiple Herpetic Epithelial Keratitis: A Case Report and Review of the Literature

**DOI:** 10.7759/cureus.61079

**Published:** 2024-05-25

**Authors:** Abdulmajeed Alkhathami, Rawan S Alsamli, Shahad A Alotaibi, Ghaida Alghamdi, Faisal Abusageah, Rana Saad Alojair, Saleh Al Othaimeen

**Affiliations:** 1 Department of Ophthalmology, College of Medicine, University of Bisha, Bisha, SAU; 2 College of Medicine and Surgery, Umm Al-Qura University, Makkah, SAU; 3 College of Medicine, Sulaiman Al Rajhi University, Al Bukayriah, SAU; 4 College of Medicine, King Saud Bin Abdulaziz University for Health Sciences, Jeddah, SAU; 5 Medicine and Surgery, Jazan University, Jazan, SAU; 6 Department of Ophthalmology, Armed Forces Hospitals Southern Region, Khamis Mushait, SAU; 7 Department of Ophthalmology, King Khaled Eye Specialist Hospital, Riyadh, SAU

**Keywords:** review of the literature, case report, atopy, herpetic epithelial keratitis, bilateral

## Abstract

Herpetic epithelial keratitis is a viral infection of the cornea caused by the herpes simplex virus (HSV). It typically presents as a unilateral disease. Bilateral involvement is a rare manifestation of herpetic epithelial keratitis, accounting for only a small percentage of cases. By sharing this case, we aim to contribute to the understanding of bilateral herpetic epithelial keratitis and stimulate further research in this area to optimize patient care and outcomes

A 13-year-old child, a known case of atopy, presented to the ophthalmology clinic with a complaint of pain, photophobia, and redness in the right eye (OD) for three days. The patient was diagnosed as a case of bilateral herpetic epithelial keratitis; he was started on moxifloxacin eye drops four times a day, Artelac (sodium hyaluronate) every two hours, carbomer HS, ganciclovir ointment five times per day.

Bilateral herpetic epithelial keratitis is a rare manifestation of HSV infection, and its management poses unique challenges compared to unilateral disease.

The diagnosis of bilateral herpetic epithelial keratitis is primarily based on clinical findings, including bilateral dendritic or geographic ulcers on the cornea. Fluorescein staining is a valuable tool for visualizing corneal ulcers. In our case, the presence of bilateral dendritic ulcers in the absence of significant anterior chamber inflammation supported the diagnosis of bilateral herpetic epithelial keratitis

Despite the limited literature on bilateral herpetic epithelial keratitis, the principles of management remain consistent with those of unilateral disease. Early recognition, prompt initiation of antiviral therapy, and close follow-up are crucial for successful outcomes.

## Introduction

Herpetic epithelial keratitis is a viral infection of the cornea caused by the herpes simplex virus (HSV). It is one of the most common causes of corneal infection worldwide, typically presenting as a unilateral disease. Bilateral involvement is a rare manifestation of herpetic epithelial keratitis, accounting for only a small percentage of cases [1]. The occurrence of bilateral disease poses unique challenges in terms of diagnosis and management.

Herpes simplex virus type 1 (HSV-1) is the most common causative agent of herpetic epithelial keratitis, although herpes simplex virus type 2 (HSV-2) can also be involved, particularly in cases of primary genital herpes. The virus is usually acquired during childhood, often through direct contact with infected secretions, such as saliva or ocular secretions, from an individual with an active orofacial or ocular herpes infection.

Herpes simplex keratitis (HSK) develops when the cornea becomes infected with HSV and is still frequently associated with unilateral corneal disease, even though the bilateral occurrence has been documented in 1.3% to 10.9% of patients [2-4]. Based on the literature, patients with atopy, long-term immunosuppression, congenital immunodeficiency, or recipients of organ transplants have a higher chance of developing bilateral HSK [2,3]. Unilateral herpetic epithelial keratitis typically presents with symptoms such as redness, tearing, photophobia, and blurred vision. The characteristic clinical findings include dendritic or geographic ulcers on the cornea, which can be visualized using fluorescein staining.

Bilateral herpetic epithelial keratitis is relatively uncommon and can occur in two distinct clinical scenarios. First, patients with a history of recurrent unilateral herpetic eye infections may develop bilateral involvement during a reactivation episode. Second, primary bilateral herpetic epithelial keratitis can occur in individuals with no prior history of herpetic eye disease [4].

The duration of treatment depends on the severity of the infection and the response to therapy. Mild cases may require several weeks of treatment, while more severe or recurrent cases may require prolonged therapy and even long-term suppressive therapy to prevent future outbreaks. Regular follow-up visits are essential to monitor the response to treatment, assess the need for adjustment of therapy, and detect any recurrence or complications.

In this case report, we present a case of bilateral herpetic epithelial keratitis. We describe the clinical presentation, diagnosis, management, and outcome of the case, highlighting the challenges encountered and the importance of early recognition and appropriate treatment. By sharing this case, we aim to contribute to the understanding of bilateral herpetic epithelial keratitis and stimulate further research in this area to optimize patient care and outcomes.

## Case presentation

A 13-year-old child, a known case of atopy, presented to the ophthalmology clinic with a complaint of pain, photophobia, and redness in the right eye (OD) for three days. The patient went to a private hospital and then he was given topical ciprofloxacin three times a day, and Tobradex ointment (tobramycin and dexamethasone) with no improvement. The patient mentioned a history of upper respiratory tract infection one week ago. The patient had a previous history of a similar attack of herpetic keratitis in the left eye (OS) two years ago (alternating). 

On examination, his visual acuity was reduced in the right eye to 20/40, and 20/20 in the left eye. Dermatological examination of the facial region revealed findings within normal limits, with no evidence of vesicular lesions observed. The findings from the slit-lamp examination are summarized in Table 1.

**Table 1:** Slit-lamp examination findings

Variable	Right eye (OD)	Left eye (OS)
Lid/ Lashes	revealed findings consistent with a normal level of cleanliness, no vesicles were seen.	revealed findings consistent with a normal level of cleanliness, no vesicles were seen.
Conjunctiva/ Sclera	Injected with mild watery, serous discharge, conjunctival follicular reaction	Conjunctival follicular reaction
Cornea	Geographic ulcer at 11 o’clock and 7 o’clock measuring 4*3.5mm OD (Fig 1), No significant stromal infiltration or endothelial involvement was noted. The ulcerative lesion did not exhibit the classic branching, dendritic patterns with terminal bulbs typically associated with herpetic keratitis. corneal sensation testing was performed and demonstrated decreased corneal sensitivity in the right eye.	nasally ghost dendrites with small vesicle lesion OS as shown in (Fig 2), However, there was an absence of any large, confluent dendritic lesions or significant corneal edema. Corneal sensation was found to be slightly decreased in the left eye as well.
Anterior chamber	Deep & Quiet	Deep & Quiet
Pupil	Regular, round, reactive	Regular, round, reactive
Lens	Clear	Clear
Fundus	Clear media, healthy disc, flat retina	Clear media, healthy disc, flat retina

**Figure 1:**
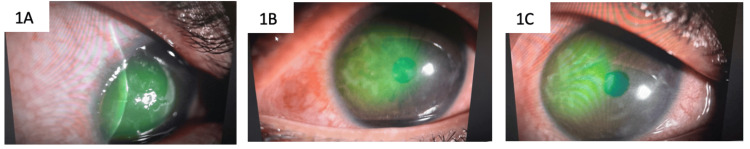
Geographic ulcer at 11 o’clock and 7 o’clock measuring 4*3.5mm in the right eye (OD)

**Figure 2:**
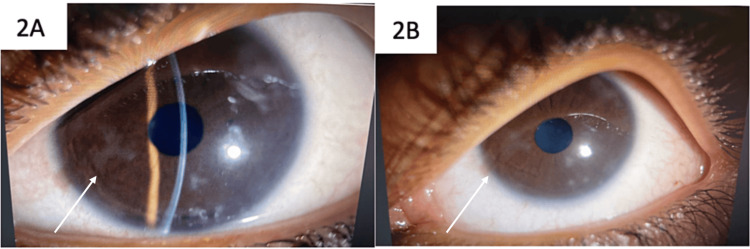
Nasally ghost dendrites with small vesicle lesion in the left eye (OS)

As depicted in Figure 1, the geographic ulcer at the 11 o’clock and 7 o’clock positions measured 4*3.5mm, providing insight into its extent in the right eye. Meanwhile, Figure 2 illustrates the presence of ghost dendrites with a small vesicle lesion nasally in the left eye

We proceeded with the microbiological analysis and scraped the ulcer's base and edges. Gram's stain and potassium hydroxide (KOH) stain were applied to corneal scraping specimens, and the results were negative in both eyes. The scrape specimen's real-time polymerase chain reaction (PCR) test revealed that both eyes had HSV-1.

Management

The patient was started on moxifloxacin eye drops four times a day, Artelac every two hours, carbomer HS, and ganciclovir five times per day. We educate the patient about the possible signs of worsening or complications, and they should seek the emergency room if they experience any sudden deterioration in vision, severe eye pain, increasing redness, or worsening symptoms despite treatment. We thoroughly educated the patient about the importance of medication compliance. We emphasized that strict adherence to the prescribed treatment regimen is essential for effective viral suppression and resolution of the herpetic keratitis infection.

Outcome

The patient showed significant improvement in symptoms and signs of infection over the following week. The visual acuity improved to 20/25 in the right eye. The corneal dendritic ulcers healed, and the epithelial erosions resolved. The patient was instructed to continue the topical and oral antiviral medications for an additional two weeks to ensure complete resolution of the herpetic keratitis infection.

Follow-up visits at regular intervals were scheduled to monitor for recurrence and assess the need for long-term suppressive therapy.

## Discussion

Bilateral herpetic epithelial keratitis is a rare manifestation of HSV infection, and its management poses unique challenges compared to unilateral disease. 

It typically presents with symptoms similar to unilateral disease, including redness, foreign body sensation, tearing, photophobia, and blurred vision. The bilateral involvement may be simultaneous or may occur sequentially, with one eye initially affected followed by the other. In our case, the patient had a history of recurrent unilateral herpetic eye infections, which puts him at a higher risk for bilateral involvement [5].

Herpetic keratitis can affect individuals of all ages, though certain age groups may exhibit distinct features and require tailored management approaches.

In pediatric patients, herpetic keratitis is often more severe, with a higher risk of vision-threatening complications such as stromal keratitis and neurotrophic ulceration. Younger children may have difficulties communicating symptoms, necessitating a high index of suspicion and thorough ophthalmic examination. Prompt initiation of antiviral therapy is crucial in this population to prevent potentially devastating outcomes [6].

In contrast, elderly patients with herpetic keratitis may present with more atypical findings, such as geographic ulceration without the characteristic dendritic patterns. Additionally, decreased corneal sensation may be less pronounced in this age group due to age-related changes in corneal innervation. Careful evaluation and a low threshold for diagnostic testing, such as corneal esthesiometry, are warranted in the geriatric population to avoid delayed diagnosis and treatment [7].

By acknowledging the varied presentations of herpetic keratitis across different age groups, the clinician can tailor the diagnostic approach and management strategies accordingly. This comprehensive understanding of the disease spectrum in pediatric, adult, and elderly patients optimizes the chances of achieving favorable outcomes for this sight-threatening condition.

The diagnosis of bilateral herpetic epithelial keratitis is primarily based on clinical findings, including bilateral dendritic or geographic ulcers on the cornea. Fluorescein staining is a valuable tool for visualizing corneal ulcers. In our case, the presence of bilateral dendritic ulcers in the absence of significant anterior chamber inflammation supported the diagnosis of bilateral herpetic epithelial keratitis [8].

Laboratory tests, such as viral culture or PCR, can be performed to confirm the diagnosis if necessary. However, it is important to note that the sensitivity of these tests may vary, and false-negative results can occur, particularly in cases of epithelial disease. Therefore, clinical judgment based on typical findings remains paramount [5].

The management of bilateral herpetic epithelial keratitis involves a combination of topical and systemic antiviral therapy. Topical antiviral agents, such as trifluridine, ganciclovir, or acyclovir ointments, are applied frequently to the affected eyes to inhibit viral replication and promote epithelial healing. Systemic antiviral medications, such as oral acyclovir, valacyclovir, or famciclovir, may be added to provide systemic suppression of the herpes simplex virus [9].

In our case, the patient was started on topical antiviral therapy and oral valacyclovir to treat the bilateral infection. The treatment duration was based on the severity of the infection and the response to therapy. Regular follow-up visits were scheduled to monitor the patient's progress and adjust therapy as needed.

The literature about bilateral herpetic keratitis is scanty, and the majority are in the form of case reports. After reviewing the literature, we provided a narrative review of the case report in the literature in Table 2. 

**Table 2:** Reported cases of bilateral herpetic keratitis in the literature.

Author/year	Patient demographic	Co-morbidities	Classic presentation	Slit-lamp findings	Treatment	Outcomes
Higaki et al. [10]	﻿37-year-old man	﻿Chronic atopic dermatitis	Decrease in vision	﻿Pustules and erosions on both eyelids, bilateral conjunctival hyperemia, and multiple dendriform corneal epithelial edema	Systemic intravenous acyclovir (750 mg/day for 7 days) for Kaposi varicelliform eruptions (KVE) and 3% acyclovir ophthalmic ointment fi ve times per day	Improved
Martone et al. [11]	﻿28-year-old man	Atopic dermatitis	﻿Progressive pain, photo- phobia, and decreased vision in both eyes	Keratitis including dendritic ulcer with underlying subepithelial infiltrate, corneal edema, and conjunctival inflammation in both eyes	﻿800 mg of acyclovir orally five times daily and with topical acyclovir ointment three times daily and tear substitutes	Completely resolved
Kitzmann AS et al. [12]	﻿77-year-old woman	﻿Pityriasis rubra pilaris (PRP)	Two-day history of severe eye pain and discharge associated with progressive visual loss in the left eye and mild foreign body sensation in the right eye.	﻿6*6-mm corneal abscess with central descemetocoele and perforation with flat anterior chamber	﻿Fortified cefazolin (50 mg/mL) and ceftazidime (50 mg/mL) every 1 hour to the left eye and oral acyclovir 400 mg 5 times daily and levofloxacin 500 mg daily + ﻿9.5-mm therapeutic penetrating keratoplasty in left eye	The best-corrected visual acuity in the left eye was 20/60. The corneal graft was clear without evidence of HSVor bacterial keratitis
Yang et al. [5]	﻿71-year-old Korean man	﻿Pemphigus foliaceus ﻿on prednisolone and azathioprine	Both eye pain and epiphora since one week	﻿Conjunctival injection and geographical corneal ulcers	Topical 3% acyclovir (Herpecid ointment five times daily, lowering the dosage of oral prednisolone to 30 mg per day and substituting oral Cyclosporin A 100 mg twice day for azathioprine	﻿The corneal edema, hypopyon, and chamber reaction of our patient improved after three weeks of systemic and topical antiviral treatment; however, the corneal epithelial defect in the left eye did not resolve, so the patient underwent amniotic membrane transplantation over the corneal epithelial defect in the left eye.
Praidou et al. [9]	﻿47-year-old female	﻿Arthritis and antinuclear antibody (ANA) positivity. Her ocular history was positive for mild anterior uveitis	﻿Bilateral redness and severe eye pain over the past week.	﻿Peripheral corneal ulcers, nummular stromal infiltrations, and central erosion of the corneal epithelium bilaterally (approximately 1.5–2 mm in diameter). Furthermore, there was a +3 inflammatory reaction of the anterior chamber in both eyes.	﻿The patient was started on oral prednisone 16 mg and cyclosporine 100 mg (daily), topical prednisolone drops (every hour), and tobramycin (4 times daily).	﻿Ten days later, after no apparent improvement, ﻿prednisone and cyclosporine were stopped, and the patient was started on topical acyclovir and systemic valacyclovir. Two weeks later, there was improvement of the epithelial defect of the right eye, total healing of the epithelial defect of the left eye
Narang et al. [13]	﻿59‑year‑old female	﻿Past history of resolved bilateral herpes simplex viral infection (HSV)	Redness, pain, and foreign body sensation in both the eyes	﻿Extensive epithelial defects, stromal edema, and bilateral circumciliary congestion	Oral valacyclovir 500 mg was prescribed three times a day, along with regular lubricants and topical antiviral treatment (ganciclovir 0.15% gel). Topical 1% prednisolone acetate was started at four times per day and reduced over a period of four weeks following the correction of epithelial abnormalities.	Within a month, the viral keratitis and ptosis completely resolved.
Agarwal et al. [14]	﻿22-year-old woman	﻿ Medically free	Sudden-onset diminution of vision associated with redness, pain and photophobia in both eyes (right>left) for 5 days	﻿A central corneal melt with an overlying 7.6×5.4 mm epithelial defect and surrounding yellowish-white stromal differential ﻿infiltrate was noted in the right eye along with diffuse congestion	Topical ointment Acyclovir 3% five times per day in both eyes and oral acyclovir at a dose of 400 mg five times per day	Therapeutic penetrating keratoplasty in the right eye and postoperatively topical prednisolone 1% 6 t/d and preservative-free lubricants were further added to the above regimen.
Mammadov et al. [15]	﻿62-year-old female	﻿Pemphigus foliaceus on ﻿peroral 40 mg methylprednisolone, topical clobetasol propionate 0.05%﻿cream, and 20 mg betamethasone valerate cream for 2 years	Burning, stinging, and redness in both eyes	Dendritic lesions in paracentral 11, 3, and 7 o’clock positions on the right cornea and in paracentral 11 o’clock position on the left cornea, mild hyperemia in the bulbar conjunctiva, and stage 2 nuclear sclerosis in both eyes.	Topical ganciclovir 5 times a day was administered for 10 days. At the same time, (iv) acyclovir 5–10 mg/kg was commenced.	Corneal lesions disappeared completely, the epithelium was intact with no stromal ulceration, and the treatment was discontinued as no further ocular findings were observed in both eyes

Despite the limited literature on bilateral herpetic epithelial keratitis, the principles of management remain consistent with those of unilateral disease. Early recognition, prompt initiation of antiviral therapy, and close follow-up are crucial for successful outcomes. Further research is needed to establish optimal treatment strategies, including the duration of therapy and the role of long-term suppressive therapy in preventing recurrences.

## Conclusions

Bilateral herpetic epithelial keratitis is a rare manifestation of HSV infection, presenting unique challenges in diagnosis and management. This case report highlighted the clinical features, diagnosis, and management of a patient with bilateral herpetic epithelial keratitis. Comparison with previously published articles underscored the importance of early recognition, prompt initiation of antiviral therapy, and regular follow-up. Further research is needed to optimize treatment strategies and improve outcomes in patients with this rare condition.
